# Accuracy of Rating Scales and Clinical Measures for Screening of Rapid Eye Movement Sleep Behavior Disorder and for Predicting Conversion to Parkinson’s Disease and Other Synucleinopathies

**DOI:** 10.3389/fneur.2018.00376

**Published:** 2018-05-25

**Authors:** Matej Skorvanek, Eva Feketeova, Monica M. Kurtis, Jan Rusz, Karel Sonka

**Affiliations:** ^1^Department of Neurology, Faculty of Medicine, P. J. Safarik University, Kosice, Slovakia; ^2^Department of Neurology, University Hospital of L. Pasteur, Kosice, Slovakia; ^3^Movement Disorders Unit, Department of Neurology, Hospital Ruber Internacional, Madrid, Spain; ^4^Department of Neurology, Center of Clinical Neuroscience, First Faculty of Medicine, Charles University and General University Hospital, Prague, Czechia; ^5^Department of Circuit Theory, Faculty of Electrical Engineering, Czech Technical University in Prague, Prague, Czechia

**Keywords:** Parkinson’s disease, RBD, idiopathic, conversion, imaging, rating scales, non-motor, synuclein

## Abstract

Rapid eye movement (REM) sleep behavior disorder (RBD) is characterized by repeated episodes of REM sleep-related vocalizations and/or complex motor behaviors. Definite diagnosis of RBD is based on history and polysomnography, both of which are less accessible due to the lack of trained specialists and high cost. While RBD may be associated with disorders like narcolepsy, focal brain lesions, and encephalitis, idiopathic RBD (iRBD) may convert to Parkinson’s disease (PD) and other synucleinopathies in more than 80% of patients and it is to date the most specific clinical prodromal marker of PD. Identification of individuals at high risk for development of PD is becoming one of the most important topics for current PD-related research as well as for future treatment trials targeting prodromal PD. Furthermore, concomitant clinical symptoms, such as subtle motor impairment, hyposmia, autonomic dysfunction, or cognitive difficulties, in subjects with iRBD may herald its phenoconversion to clinically manifest parkinsonism. The assessment of these motor and non-motor symptoms in iRBD may increase the sensitivity and specificity in identifying prodromal PD subjects. This review evaluates the diagnostic accuracy of individual rating scales and validated single items for screening of RBD and the role and accuracy of available clinical, electrophysiological, imaging, and tissue biomarkers in predicting the phenoconversion from iRBD to clinically manifest synucleinopathies.

## Introduction

Rapid eye movement (REM) sleep behavior disorder (RBD) belongs to the parasomnias, which is a group of disorders characterized by paroxysmal motor and behavioral events occurring exclusively during sleep. RBD is defined by dream enactment and complex motor behaviors during REM sleep and loss of normal REM sleep muscle atonia (also known as REM sleep without atonia—RSWA) as detected by polysomnography (PSG) (1). It is often associated with frightening dreams and may result in sleep-related injuries to the patients as well as to their sleeping partners (2). RBD can also be differentiated as symptomatic and idiopathic. Symptomatic RBD has been previously linked to several etiologies, especially synucleinopathies and narcolepsy, but also to brain lesions, autoimmune, and inflammatory disorders (3–5).

Several reports have shown, that idiopathic RBD (iRBD) may convert to Parkinson’s disease (PD) or other synucleinopathies, including multiple system atrophy (MSA) and Dementia with Lewy Bodies (DLB), in more than 80% of patients (6, 7). While specificity of iRBD in identification of prodromal PD subjects seems to be very high, its prevalence in the general population is rather low and was reported in the range of 0.3–1.15% when confirmed by video-PSG in populations older than 60 years (2, 8). This prevalence may, however, be underreported as patients with mild severity of symptoms and those without sleeping partners are less likely to report their symptoms and seek medical care. On the other hand, various sleep disorders, such as severe obstructive sleep apnea (OSA), periodic limb movements (PLM), sleepwalking and others, may mimic RBD symptoms and may result in false-positive screening in questionnaire-based population studies. The definite diagnosis of RBD is based on history and PSG (1), both of which are less accessible due to the lack of trained specialists and cost. Thus, several instruments have been developed to enable large population screening and selection of candidates for more detailed testing in sleep centers. However, their diagnostic accuracy seems to depend on the studied population and instrument used and no clear recommendations have been made to this date regarding their use in routine clinical practice.

Parkinson’s disease and other synucleinopathies are complex progressive multiorgan disorders affecting different neurotransmitter systems across the central, peripheral, and autonomic nervous systems, with Lewy bodies, Lewy neurites or glial cytoplasmic inclusions as their pathological hallmark (9, 10). In addition to iRBD, other well-recognized non-motor symptoms (NMS), such as hyposmia, constipation, or mood disorders; subtle motor abnormalities; and/or imaging findings may precede the onset of clinically relevant motor or cognitive symptoms by years or even decades (11). These known risk and prodromal markers have been recently summarized in the International Parkinson and Movement Disorder Society (MDS) research criteria for prodromal PD (11) and other novel biomarkers are still emerging. iRBD seems to be the most specific of these clinical prodromal markers so far and thus an optimal candidate for identifying patients for future disease-modifying and neuroprotective treatments. However, before these criteria can enter routine clinical practice, several key issues need to be addressed. These include the development of efficient tools for screening and identification of RBD subjects in the general population and the identification of other measures enabling accurate prediction and estimation of the time frame of phenoconversion from iRBD to a clinically manifest synucleinopathy.

Thus, the aim of this review is to summarize the current evidence on the diagnostic accuracy of screening instruments for identification of iRBD and to summarize the available data on other symptoms and signs, including presence of subtle motor dysfunction, NMS, neuroimaging findings, electrophysiological and tissue biomarkers, in predicting the phenoconversion of iRBD to PD and other synucleinopathies.

## Methods

We searched PubMed for all relevant articles until October 2017, using main search terms “RBD” or “REM sleep behavior disorder” combined with terms: “idiopathic,” “Parkinson*,” “DLB,” “MSA,” “synuclein*,” “screen*,” “questionnaire,” “scale,” “inventory,” “accuracy,” “sensitivity,” “specificity,” “conversion,” “predict*,” “motor,” “UPDRS,” “speech,” “gait,” “non-motor,” “NMS,” “olfactory,” “hyposmia,” “autonomic,” “cardial,” “urinary,” “constipation,” “sleepiness,” “EDS,” “cognit*,” “imaging,” “DaT,” “PET,” “MRI,” “sonography,” “SPECT,” “substantia nigra,” “serum,” “CSF,” “cerebrospinal,” and “biopsy.” Only studies in English language and related to human studies were included. All titles and abstracts were reviewed for relevance. References were supplemented by selection from the reference lists of identified papers, as well as personal knowledge of emerging literature.

## Accuracy of Screening Instruments for RBD

Definite diagnosis of RBD is based on PSG, which demands specific training, is costly and not widely available. Therefore, several screening instruments have been developed to enable screening for RBD. Four RBD-specific questionnaires, two single item RBD-screening questions, and two generic instruments containing items on RBD were identified and discussed below by order of available clinimetric data. The RBD severity scale was not included in this review, since it is based on rating of PSG recordings, and thus does not qualify as a screening tool for RBD *per se* (12).

### REM Sleep Behavior Disorder Questionnaire Hong Kong (RBDQ-HK)

#### Scale Description

The RBDQ-HK was developed to evaluate the presence, but also the frequency and severity of RBD (13). A modified version with added screening questions on severe OSA (14) and validated language translations are available (15). The scale covers occurrence and frequency of dreams and nightmares, dream content, vocalizations during sleep, motor behaviors during sleep, injuries during sleep, and sleep disruptions. The RBDQ-HK is composed of 13 items, each assessing two dimensions—(a) lifetime occurrence (don’t know, no, yes) and (b) recent 1-year frequency (once or few times per year; once or few times per month, 1–2 times per week, 3 times or above per week). Questions are weighted differentially and range from 0 (no RBD symptoms) to 100 (highest severity of RBD symptoms), based on the score for lifetime occurrence (0–20) and recent 1-year frequency (0–80). Factor analysis resulted in a significant two-factor solution, factor 1 being dream related (items 1–5 and 13, score range 0–30) and factor 2 evaluating behavioral manifestations (items 6–12, score range 0–70). Administration time is typically up to 15–20 min. The scale is of public domain.

#### Clinimetric Properties

The scale has shown good reliability with Cronbach’s alpha of 0.74–0.90 and test–retest intraclass correlation coefficients of 0.80–0.92 (13, 15, 16). Construct validity was good with a value of Kaiser–Meyer–Olkin’s measure of sampling adequacy of 0.89–0.91 and Bartlett’s test of sphericity which yielded a significance value of less than 0.001, rendering an exploratory factor analysis adequate for the RBDQ-HK questionnaire (13, 15). The RBDQ showed moderate correlation with the RBD-screening questionnaire (*r* = 0.51, *p* < 0.01) and low correlation with the RBD severity scale (*r* = 0.35, *p* < 0.01) (15). Sensitivity to change after treatment was demonstrated in multiple studies (17, 18).

#### Score Differences in Studied Populations

RBDQ-HK scores did not differ between early- and late-onset RBD (19) and also between subjects with iRBD, symptomatic RBD and RBD-like disorders due to psychotropic medications or psychiatric illness (13). Males were found to have significantly higher scores for behavioral manifestations (factor 2) (*p* = 0.019), with more dream-related movements and falling out of bed compared to females (20). RBDQ-HK scores in subjects with RBD and dementia were found to be significantly higher (*p* = 0.029) compared to RBD patients without dementia (21).

#### Diagnostic Accuracy

There are four studies that have evaluated the diagnostic accuracy of the RBDQ in PSG-confirmed RBD populations with acceptable to excellent results for diagnostic accuracy in the general RBD population with iRBD, PD-related RBD and RBD-like disorders due to psychotropic medications and psychiatric illness (13–16) (see Table 1). The sensitivity and positive predictive value (PPV) of RBDQ-HK were lower in OSA patients. Shen et al. (16) reported superior diagnostic accuracy of Factor 2 score compared to total RBDQ score in their total RBD sample as well as in PD and OSA patients. Also, adding two items related to OSA in the modified RBDQ-Beijing scale (14) resulted in improved specificity and PPV, while excellent sensitivity and negative predictive value (NPV) were retained.

**Table 1:** Diagnostic accuracy of the rapid eye movement (REM) sleep behavior questionnaire (RBDQ-HK).

Reference	Study population	No of subjects	Mean values	Proposed cutoff	Sensitivity (%)	Specificity (%)	PPV (%)	NPV (%)	AUC	Comments
Li et al. (13)	Total RBD sample	107	32.1 ± 16.1	18/19	82.2	86.9	86.3	83.0	0.90	*Mostly associated with psychotropic medications and psychiatric illness **RBDQ-HK items 6–12—behavioral manifestations, score range 0–70
	iRBD	51	29.7 ± 15.5	18/19	78.4	86.9	74.1	89.4	0.89	
	sRBD (mostly PD)	29	30.8 ± 17.3	18/19	79.3	86.9	62.2	93.9	0.88	
	RBD-like disorders*	27	38.1 ± 14.9	20/21	92.6	86.9	64.1	98.0	0.94	
	Factor 2 score**	107		7/8	87.9	81.3	82.5	87.0	0.92	
	Controls	107	9.5 ± 10.2							

Sasai et al. (15)	iRBD Controls	122	46.4 ± 12.6	19/20	97.2	97.5	97.5	97.2	0.99	
		106	5.7 ± 6.8							

Shen et al. (16)	All RBD	115								**RBDQ-HK items 6–12—behavioral manifestations, score range 0–70
	Total score		38.4 ± 21.7	17	84.4	81.0	70.8	90.4	0.89	
	Factor 2 score**			7/8	90.4	82.2	73.8	94.0	0.91	
	PD patients	95	32.6 ± 23.1							
	With RBD	61								
	Total score		42.1 ± 21.5	18	86.9	70.6	81.8	75.8	0.84	
	Factor 2 score**			13	83.6	76.5	87.4	65.6	0.86	
	Without RBD	34	15.7 ± 15.0							
	OSA patients	144	12.0 ± 13.2							
	With RBD	30								
	Total score		26.3 ± 18.1	17	70.0	86.8	52.5	91.4	0.85	
	Factor 2 score**			7	83.3	87.7	64.1	95.2	0.88	
	Without RBD	114	8.2 ± 8.3							

Chang et al. (14)	All RBD	118								***Modified version RBDQ-Beijing includes 2 additional items on OSAS, scores range 0–110
	RBDQ-HK	118		18/19	97.1	83.2	86.4	96.3		
	RBDQ-Beijing***	118		28/29	95.8	94.3	95.0	95.2		
	Controls	106								

*PPV, positive predictive value; NPV, negative predictive value; AUC, area under curve; iRBD, idiopathic REM sleep behavior disorder; sRBD, symptomatic REM sleep behavior disorder; PD, Parkinson’s disease; OSAS, obstructive sleep apnea syndrome; OSA, obstructive sleep apnea*.

#### Strengths and Limitations

The RBDQ-HK is a well validated instrument with good psychometric properties and good diagnostic accuracy. Compared to some other RBD-screening instruments it evaluates not only presence, but also frequency and severity of RBD symptoms historically and more recently. On the other hand, due to its relatively longer administration time, it is less suitable for first line screening in large population-based studies. Although the English version of the scale has been published, it seems that it was not translated systematically, was not validated, and thus has some cultural specific phrases (e.g., chased by a ghost) that may not be appropriate in non-Chinese populations (22).

### REM Sleep Behavior Disorder Screening Questionnaire (RBDSQ)

#### Scale Description

The RBDSQ was developed as a simple screening tool for RBD (23). The scale was originally developed in English and German, but currently several validated language translations are available (24–27). The RBDSQ covers frequency, dream content, nocturnal movements, injuries to self or bed partner, types of motor behaviors during the night, nocturnal awakenings, sleep disruption, and the presence of neurological diseases. It is comprised of 13 yes/no questions. Total scores range from 0 (no symptoms) to 13 (maximum score). Administration time is typically 5 min. The scale is of public domain.

#### Clinimetric Properties

The scale has shown good reliability with Cronbach’s alpha of 0.77–0.89 (23, 25) and test–retest intraclass correlation coefficients of 0.84–0.95 (24, 26). In the original validation cohort, the item test correlations ranged from 0.34 (item 9) to 0.75 (item 6.1) (acceptable if > 0.30). In the Italian version of RBDSQ item 10 had a low item-total correlation (0.14) (27). Regarding convergent validity, the RBDSQ showed moderate correlations with the RBDQ-HK (*r* = 0.51, *p* < 0.001) (15). On the other hand, significant correlations with the PLM index, number of PLM during sleep and PLM during wakefulness was found in a cohort suffering with other sleep disturbances (23). Sensitivity to change after treatment has not been studied. Stefani et al. (28) have shown an increase of RBDSQ score by 1 point (*p* < 0.001) in the general population over 2 years, however, the consistency of score changes over this period was low (Spearmen *r* = 0.35; ICC 0.39).

#### Score Differences in Studied Populations

RBDSQ scores did not correlate with age (28) or duration of RBD (23). There were no gender differences between RBDSQ total scores, nevertheless, men had more fights, violent behaviors, and awakenings by own movement, while women experienced more disturbed sleep (29). The mean RBDSQ scores of subjects with iRBD were similar to those with symptomatic RBD, but significantly higher compared to patients with other sleep disturbances, PD without RBD or healthy controls (25). The scores of RBDSQ in patients with other sleep disturbances were highest for subjects with sleepwalking and epilepsy, followed by narcolepsy, insomnia, PLM, restless legs (RLS) or hypersomnia. Scores of RBD patients with and without narcolepsy did not differ (23).

#### Diagnostic Accuracy

There are nine studies that have evaluated the diagnostic accuracy of the RBDSQ in PSG-confirmed RBD populations with sensitivity ranging from moderate to excellent and specificity ranging from low to excellent based on study population and cutoff used (see Table 2). While both sensitivity and specificity were good to excellent when comparing RBD subjects with healthy controls, the specificity was generally lower when including other sleep disorders in the control groups. Stiasny-Kolster et al. (30) have shown that administration of the RBDSQ in randomly selected PD patients without prior sleep interviews yielded only moderate sensitivity and specificity as opposed to a PD cohort which was specifically selected for validation of the RBDSQ and included a sleep interview prior to administration of the questionnaire. Mixed specificity was found in studies evaluating diagnostic accuracy of RBDSQ in OSA (24, 26). Using a cutoff value of 5, a recent meta-analysis found pooled sensitivity, specificity, positive likelihood ratio (LR), negative LR, and diagnostic odds ratio (OR) of RBDSQ to be 0.91 (95% confidence interval (CI) 0.85–0.95), 0.77 (95% CI 0.66–0.85), 4.00 (95% CI 2.60–6.10), 0.12 (95% CI 0.07–0.19), and 34 (95% CI 16–71), respectively (31).

**Table 2:** Diagnostic accuracy of the Rapid Eye Movement (REM) sleep behavior screening questionnaire (RBDSQ).

Reference	Study population	No of subjects	Mean values	Proposed cutoff	Sensitivity (%)	Specificity (%)	PPV (%)	NPV (%)	AUC	Comments
Stiasny-Kolster et al. (23)	RBD	54	9.5 ± 2.8							*Including other sleep-related distrubances
	All controls*	160	4.6 ± 3.0	5	96	56	–	–	0.87	
	Healthy controls	133	2.0 ± 1.8	5	96	92	–	–		

Marelli et al. (27)	All RBD	76	9.8 ± 2.3				–	–	0.89	*Including other sleep-related disturbances **without item 10
	All Controls*	405*	5.2 ± 2.8	8	84	78	–	–		
				5	97	46	–	–	0.90	
	Modified RBDSQ**			8	83	82				

Wang et al. (25)	iRBD	41	8.1 ± 2.7							
	Other sleep abnormalities	78	3.2 ± 1.7	5	90.2	82.1	–	–	0.93	
	Healthy controls	50	2.3 ± 2.0	5	90.2	76.0	–	–	0.95	
	sRBD	22	8.0 ± 2.0	6	90.9	91.9	–	–	0.97	

Miyamoto et al. (24)	iRBD	52	7.5 ± 2.8							
	Healthy controls	65	1.6 ± 1.2	4.5	88.5	96.9	97.9	91.4	0.97	
	OSA	55	1.9 ± 2.3	4.5	88.5	90.9	90.2	89.3	0.93	

Comert et al. (26)	RBD	17	9.4 ± 1.6							
	OSA	28	4.1 ± 2.9	5	100	64	63	100	0.92	
	Healthy controls	78	2.9 ± 2.2	5	100	87	63	100	0.97	

Lee et al. (32)	iRBD	47	9.0 ± 2.0							*Newly diagnosed and untreated, patients with other sleep disturbances excluded
	OSA	213*	2.0 ± 3.0	6.5	85.1	93.4	74.1	96.6	0.92	
				4.5	89.4	77.5	46.7	97.1		
	Healthy controls	58	1.0 ± 3.0	4.5	89.4	98.3	97.7	91.9	0.99	

Chahine et al. (33)	PD with RBD									
	PD without RBD			7	74.2	82.4			0.80	

Nomura et al. (34)	PD with RBD	19	7.2 ± 1.9							All RBD versus non-RBD
	PD without RBD	26	2.9 ± 1.6	6	84.2	96.2				
	iRBD	31	7.9 ± 2.8							

Stiasny-Kolster et al. (30)	PD with RBD (A)	37	7.5 ± 2.4	5	90.0	87.0			0.95	A—PD sample specifically selected to validate RBDSQ including prior interview B—randomly selected PD patients without prior interview
	PD without RBD (A)	15	3.1 ± 1.4	6	78.0	100				
	PD with RBD (B)	56	6.0 ± 3.1	5	68.0	63.0			0.67	
	PD without RBD (B)	19	4.2 ± 2.7	6	64.0	68.0				

*PPV, positive predictive value; NPV, negative predictive value; AUC, area under curve; iRBD, idiopathic REM sleep behavior disorder; sRBD, symptomatic REM sleep behavior disorder; PD, Parkinson’s disease; OSA, obstructive sleep apnea*.

#### Strengths and Limitations

The RBDSQ is currently the most commonly used screening instrument for RBD, with short administration time, good reliability and overall good diagnostic accuracy in the general population. However, different aspects of validity have not been properly studied, sensitivity to change after treatment has not been demonstrated and its diagnostic accuracy in specific populations, such as subjects with other sleep disorders or in randomly selected PD patients without prior interview, was less satisfactory (31).

### Mayo Sleep Questionnaire (MSQ)

#### Scale Description

In 2001, two versions of the MSQ were originally developed, one to be filled out by the patient and the other by his/her bedside partner/informant. Pilot data showed that the informant sleeping in the same room provided information with higher sensitivity and specificity than the patient (regardless of patient cognitive status) when correlated with PSG (35). Thus, the partner/informant version is currently the most widely used. It is a 16-item scale that screens for the presence of RBD but also for periodic leg movements (PLM), RLS, sleep walking, OSA and sleep-related leg cramps. There is one sole screening question for RBD (question 1) which asks about acting out dreams (punching/screaming) and is answered yes or no if the behavior has happened at least three times (time frame is since you remember). If yes, the partner continues to answer the subsequent 5 items (a–e). The questionnaire can be completed in about 2 min and is of public domain.

#### Clinimetric Properties

Published data on reliability, validity, with exception of criterion validity (see diagnostic accuracy below), and sensitivity to change are not available.

#### Diagnostic Accuracy

Question 1 of this questionnaire has been found to have an excellent sensitivity and acceptable to excellent specificity for determining the presence of RBD when compared to PSG results (36, 37) (see Table 3). Four additional questions on dream enactment leading to patient or partner injuries, dream content and its correlation with movements, improved specificity (36). In further studies, Chahine et al. (33) and Bolitho et al. (38) diagnosed RBD in PD populations based on PSG and contrasted results with several RBD questionnaires, including the MSQ (see Table 3), which showed high sensitivity but low specificity.

**Table 3:** Diagnostic accuracy of other RBD rating scales and screening items.

Scale	Reference	Study population	No of subjects	Proposed cutoff	Sensitivity	Specificity	PPV	NPV	AUC	Comments
MSQ	Boeve et al. (36)	Aging dementia cohort	176	Yes (core question 1)	98	74				

MSQ	Boeve et al. (5, 37)	Community based (normal, MCI, AD)	95		100	95				

MSQ	Chahine et al. (33)	PD	75	Yes/no question 1	90.3	54.6			0.7	69.3% correctly classified. 2.0 (1.4–2.8) + LR

MSQ	Bolitho et al. (38)	PD	46/31 with bed partners	Yes (core question 1)	95	64	83	88		vs REM EMG density
					100	36	43	100		vs REM atonia index

RBD-I	Frauscher et al. (39, 40)	RBD (idiopathic, PD, narcolepsy, other) and controls	210	0.25	91.4	85.7			0.886	

IRBDIQ	Frauscher et al. (39, 40)	Same as above	210	Single item	74.3	92.9			0.836	

RBD1Q	Postuma et al. (21, 41, 42)	iRBD	242	Single item	93.8	87.2	87.9	93.4	0.905	
		Controls—healthy subjects, OSA, RLS, other	242							

RBD1Q	Bolitho et al. (38)	PD with/without RBD	46	Single item	100	48	48	100		vs REM atonia index
					93	68	81	87		vs REM EMG density

RBD1Q	Ma et al. (43)	iRBD	14	Single item	100	55.6	63.6	100	0.778	
		Controls	18							

*PPV, positive predictive value; NPV, negative predictive value; AUC, area under curve; MSQ, Mayo Sleep Questionnaire; MCI, mild cognitive impairment; AD, Alzheimer disease; PD, Parkinson disease; +LR, positive likelihood ratio; REM, rapid eye movement; EMG, electromyography; RBD-I, Innsbruck RBD inventory; IRBDIQ, Innsbruck RBD Inventory Single Question; RBD1Q, RBD single-question screening; OSA, obstructive sleep apnea; RLS, restless legs syndrome; iRBD, idiopathic RBD*.

#### Score Differences in Studied Populations

This questionnaire has been used by the group that developed it in two studies with aging and predominantly male populations (mean age 71, median age 77, respectively) (36, 37). The earlier publication studied an aging and dementia cohort (controls = 8, MCI = 44, AD = 23, DLB = 74, dementia with parkinsonism other = 27) where 55% had RBD (36). The second studied a community-based cohort with mostly cognitively normal subjects and only 5% were found to have RBD (37).

#### Strengths and Limitations

The questionnaire is simple and easy to complete. However, it is limited to patients with a partner that sleeps in the same room or has slept in the same room. The MSQ has a single question that screens for RBD, clinimetric properties of the questionnaire and this single question require further study.

### Innsbruck RBD Inventory (RBD-I)

#### Scale Description

The Innsbruck RBD-I (39) is a short screening questionnaire for RBD made up of five questions. The questions are answered yes (1), no (0), or don’t know. The RBD symptom score is equal to the number of positive answers divided by the number of answered questions and thus ranges between 0 and 1. The second part of the questionnaire determines frequency of behavior and ranges from never (0) to very frequent (4). RBD frequency score is the sum of all frequency scores divided by the number of questions answered, thus ranging from 0 (minimum) to 4 (maximum) and may give information about severity. It can be completed in a few minutes. There is an English and a German version.

#### Clinimetric Properties

Cronbach’s alpha for RBD-I was 0.855, test-retest reliability was not assessed (39). Correlation between RBD frequency score and patient global impression was 0.305 (*p* = 0.011) (39). There is no data available on sensitivity to change.

#### Diagnostic Accuracy

This inventory was validated in a population of 70 RBD subjects and 140 controls. Originally the questionnaire had 7 REM sleep-related questions and 2 non-REM behavior control items (sleep walking, snoring). Only the response patterns to the REM sleep items discriminated between patients and controls (*p* < 0.05). Five of the 9 items had an AUC > 0.7 and were thus included in the final version of the questionnaire (See Table 3). The sensitivity and specificity of RBD-I in a single cohort of clinically diagnosed iRBD patients versus healthy controls reporting on diagnostic accuracy was good-excellent (39).

#### Score Differences in Study Populations

In the validation study, there were no differences in scores between the PD population and the iRBD group nor between patients with or without bed partners (39).

#### Strengths and Limitations

This simple and easy to complete questionnaire can be used for screening and may give information about RBD severity based on the frequency score. However, the original study was performed on a population that had an RBD diagnosis based on PSG which may have influenced questionnaire completion. Further validation and reliability data and data from other groups beyond the developers are needed.

### Single RBD-Screening Questions

#### REM Sleep Behavior Disorder Single-Question Screening (RBD1Q)

The RBD1Q was developed as a simple screening tool for large-scale epidemiological studies (41) It consists of a single question, answered “yes” or “no,” as follows: “Have you ever been told, or suspected yourself, that you seem to ‘act out your dreams’ while asleep (for example, punching, flailing your arms in the air, making running movements, etc.)?” This question was initially translated into French, German, Japanese, Italian, Spanish, Czech and Danish, and subsequently also in multiple other languages. The screen was designed to be self-administered, with participation by spouses/caregivers encouraged. If literacy was poor, administration by rater was allowed. The RBD1Q is of the public domain.

##### Diagnostic Accuracy

The sensitivity and specificity of RBD1Q was found good-excellent compared to controls and OSA in the initial validation study of iRBD patients and the sensitivity and specificity were not significantly altered after excluding patients who lived alone, were using antidepressants, clonazepam, or melatonin (41). While sensitivity was generally excellent across other studies as well, the specificity of RBD1Q was found low to moderate when evaluating RBD1Q against PSG criteria in other studies (38, 43) (see Table 3).

##### Strengths and Limitations

The RBD1Q is the most commonly used single question screening instrument for RBD with a good-excellent sensitivity across multiple studies, and thus a good candidate for first step screening in large-scale population studies. Nevertheless, it does not screen for sleep talking and sleep yelling, which might be the only manifestations of RBD in a subset of patients and may be, therefore, missed on screening. Also the specificity of the scale was found rather low in multiple studies.

#### Innsbruck RBD-I Single Question (IRBDIQ)

The same group that developed the RBD-I (39) also assessed the value of a single RBD summary question: “Do you hit or kick because you dream that you have to defend yourself?” Based on this yes or no answer, the patient is classified as probable RBD or not.

##### Diagnostic Accuracy

This single item showed low sensitivity and high specificity in the original validating study (see Table 3).

##### Strengths and Limitations

The single question asks about violent dream enactment behaviors and thus has a high specificity for RBD. However, it may miss milder cases showing a relatively moderate sensitivity. Therefore, this summary question is not recommended for screening in the general population but may prove useful in population-based neurodegenerative risk-marker studies.

### Generic Rating Scales Including RBD-Related Items

#### The Non-Motor Symptom Questionnaire (NMQuest)

##### Scale Description

The NMSQuest (44) is a PD-specific global scale to screen for NMS. It is made up of 30 yes/no questions covering 10 domains: cardiovascular (2 items), gastrointestinal (8), urinary (2), sexual (2), sleep/fatigue (5), sudomotor (1), and miscellaneous (10). Two items refer to RBD-related symptoms referring to the presence of vivid or frightening dreams (question 24) and, more specifically, to moving in dreams as if acting them out (question 25). It is a patient reported outcome and can be completed in 5–10 min.

##### Clinimetric Properties

The original publication demonstrated good validity, feasibility and acceptability with low ceiling and floor effects (44). Further testing showed moderate average sensitivity for all items (63.4%) and high mean specificity for most items (88.5%) (45). As far as RBD, this study used a semi-structured clinical interview for RBD diagnosis and found that item 25 had a 69.7% sensitivity, 91.4% specificity, and PPV of 88.5% and NPV of 76.2%.

##### Strengths and Limitations

This questionnaire can be recommended for screening NMS in PD and has been proposed to evaluate burden of NMS as a whole (46). The MDS task force recommended or suggested the NMSQuest for screening of some non-motor domains such as gastrointestinal, urinary and orthostatic symptoms (47). The role of the two sleep items on the NMSQuest alluding to possible REM sleep disturbances in screening for RBD requires further testing and comparison to polysomnographic results. Therefore, the diagnostic value of NMSQuest in screening for RBD or for predicting conversion to synucleinopathies is not known at this moment.

#### The Movement Disorders Non-Motor Symptom Scale (MDS-NMSS)

The MDS-NMSS is currently in the process of validation (48). This revised version of the Non-Motor Symptom Scale (NMSS) is designed to include one item in the sleep wakefulness domain that alludes to RBD symptoms. Supposedly, the clinician will ask the patient and his partner about acting out dreams while asleep, such as shouting, flailing arms, punching, or running movements. As in the original NMSS, the time frame will be the last month and the rater will evaluate for presence of the symptom and its frequency. The addition of this item to the NMSS demonstrates the importance of RBD as a salient NMS in PD.

## Diagnostic Accuracy of Clinical Measures for Predicting Conversion to PD and Other Synucleinopathies

### Subtle Motor Impairment

Among different prodromal markers, subtle motor dysfunction strongly predicts conversion from iRBD to degenerative parkinsonism, regardless of primary diagnosis of PD or DLB (49). However, studies investigating different aspects of subtle motor changes in iRBD are rather scarce. Most evidence is based on a cross-sectional study comparing results of 68 iRBD subjects to 36 controls, 34 PD patients with RBD and 21 PD patients without RBD using clinical scales and quantitative motor testing (50). In addition, a follow-up longitudinal study investigated motor changes in 20 subjects with iRBD who developed parkinsonism and their motor testing results from the preceding 5 years were assessed with regression analysis to determine when clinical markers first deviated from normal values (42). Pilot results regarding instrumental assessment of speech and gait dysfunction in iRBD have also been published (51–54).

#### Unified Parkinson’s Disease Rating Scale (UPDRS)/Movement Disorder Society-Unified Parkinson’s Disease Rating Scale (MDS-UPDRS)

Although examination using the UPDRS is rather subjective and requires a well-trained examiner, it still represents the most widely used instrument for measuring severity of parkinsonian symptoms with the motor part allowing assessment of the cardinal manifestations of PD including bradykinesia, rigidity, resting tremor and gait abnormalities (55). The MDS-UPDRS was developed to address the identified shortcomings of the original UPDRS (56). It discriminates the slight and mild manifestations of the disease making the scale preferable in early stages of disease, provides uniform item scoring and clear instructions to both raters and patients (56). Higher UPDRS part III (motor examination) scores associated with increased risk of parkinsonism were observed in iRBD compared to controls (42, 50). In particular, higher UPDRS part III scores have been estimated to deviate from normal values approximately 4.5 years before diagnosis (42). The diagnostic cutoff value was identified as UPDRS part III >3 points and MDS-UPDRS part III (motor examination) >6 points with removal of action tremor, which is common in non-parkinsonian conditions (11, 42). Such scores on the UPDRS 2 years prior showed a very high specificity of 97% and sensitivity of 94% with overall area under curve (AUC) of 0.99 for conversion to parkinsonism (42),. These cutoffs, however, still need to be validated in more prospective trials, as up to 30% of elderly patients may score >6 points on the MDS-UPDRS part III without including the action tremor items (57).

#### Hand Motor Speed Tests

Upper limb movements in iRBD possibly present with both rigidity and bradykinesia. Indeed, UPDRS subscores on rigidity and limb bradykinesia deviated from normal values at estimated intervals of 4.4 and 4.2 years before diagnosis, respectively (42). In addition, patients with iRBD showed deteriorated performance in Purdue pegboard and alternate tap tests compared to controls (50). Scores on these hand motor speed tests have been estimated to deviate from normal values at 8.6 and 8.2 years before diagnosis of PD, respectively (42). Using the Purdue pegboard and alternate tap tests, parkinsonism in iRBD patients may be detected 3 years before clinical diagnosis with a 71*–*82% sensitivity and specificity (AUC = ~0.80) (42). These findings need to be reproduced in an independent population.

#### Speech Impairment

Based upon UPDRS scores, longitudinal changes in items evaluating voice/face in an iRBD population were estimated as the first motor signs to develop 9.8 years before diagnosis followed by rigidity, gait abnormalities, and limb bradykinesia (42). The assumption that speech impairment in iRBD represents a sensitive prodromal marker of neurodegeneration was consequently confirmed by a pilot cross-sectional study comparing vocal performance in 16 iRBD patients and 16 controls using quantitative and objective acoustic voice analysis (54). Some form of speech impairment was observed in 88% of iRBD subjects, leading to a promising sensitivity of 96% and specificity of 79% (AUC = 0.83) in distinguishing between iRBD and control subjects (54). Subsequently, automated analysis of connected speech revealed similar speech timing deficits in independent cohorts of 50 iRBD and 30 *de novo* PD patients (53). The main speech abnormalities found in iRBD were prolonged duration of pauses, longer length of stop consonants and decreased rate of switching between follow-up speech segments (53). In general, speech disorders were more prominent in iRBD subjects with higher motor scores on the UPDRS (53, 54), suggesting that speech impairment partially parallels increasing motor disability due to the underlying neurodegenerative process. Nevertheless, the potential predictive value of instrumentally assessed vocal abnormalities needs to be established in prospective follow-up studies.

#### Gait Dysfunction

Gait abnormalities in iRBD deviated from normal values at an estimated interval of 4.4 years based on the UPDRS and 6.3 years using the timed up-and-go test before formal diagnosis of PD (42). Timed up-and-go test statistically separated iRBD from controls (50) and was able to distinguish both groups with a high sensitivity 90% but low specificity 46% (AUC = 0.71) 3 years before conversion of the subject with iRBD to parkinsonism (42). Based on automated gait analysis (GAITRite) collected from 42 probable RBD subjects and 492 controls, presence of RBD was associated with decreased velocity and cadence, increased double limb support variability, and greater stride time variability and swing time variability (51). Furthermore, in a pilot study based on four groups of 10 participants, significant reductions were seen in the posterior shift of the center of pressure during the propulsive phase of gait initiation in the iRBD and PD with freezing of gait patients compared to controls and PD subjects without freezing of gait (52). These reductions negatively correlated with the amount of REM sleep without atonia (52). Longitudinal studies are necessary to estimate reliability of instrumental gait analysis to predict conversion from iRBD into parkinsonism.

##### Conclusion

Motor abnormalities are very strong predictors of conversion to synucleinopathy with one of the greatest hazard ratios (HR) of 3.9 among available predictive markers to date (49). However, no consensus exists on the ideal method for examination of these subtle motor changes. For example, no agreement has been reached whether to use traditional scales or instrumental devices or whether to focus on movement velocity or amplitude. Several objective quantitative methods for testing motor function are being developed. In particular, assessment of *s*peech and gait dysfunction seem to have potential to provide sensitive motor markers of prodromal neurodegeneration; however, longitudinal studies are necessary to estimate predictive values of these examinations.

### Non-Motor Symptoms

Multiple prodromal clinical NMS have been studied in iRBD patients to identify subjects with an increased risk and time frame of conversion to synucleinopathy. Most of these NMS are recognized as risk or prodromal markers for development of PD (11).

#### Olfactory Loss

Several studies have shown increased prevalence of olfactory impairment in iRBD subjects compared to healthy controls and similar, or slightly lower, prevalence compared to clinically diagnosed PD patients (50, 58–61). In one study (62), 9 of the 34 iRBD patients converted over a 5-year follow-up period, 6 to PD, 3 to DLB, and significantly higher prevalence of olfactory dysfunction was found in converters compared to non-converters. The Sniffin’ Sticks test showed a diagnostic accuracy of 82.4% in predicting conversion after 2.4 ± 1.7 years. Similarly, Fereshtehnejad et al. (63) found a significantly higher prevalence of olfactory dysfunction, measured by the UPSIT-12, in iRBD subjects who converted compared to those who did not convert to synucleinopathy (75.0 vs 46.6%). In this study, prodromal NMS appeared to be largely independent of one another while interaction between hyposmia and quantitative motor testing was found. In this regard, abnormal baseline quantitative motor testing increased conversion only in subjects who also had hyposmia (OR = 10.0; 95% CI: 3.3–30.3; *p* < 0.001), with no predictive value in those with normal olfaction (OR = 1.1; 95% CI: 0.2–6.3; *p* = 0.91) (63). Mean disease-free follow-up in this study was 3.6 years, while total follow-up duration was 5.7 years. On the other hand, in another iRBD cohort, Li et al. (64) in their iRBD cohort found slightly worse olfaction scores in converters compared to non-converters, this was not statistically significant (HR = 1.22; adjusted 95% CI: 0.48–3.11).

##### Conclusion

Olfactory loss is significantly higher in iRBD subjects compared to healthy controls, severity is reported to be intermediate between controls and PD patients. Olfactory dysfunction may predict early conversion to clinically manifest synucleinopathy and seems to be independent from other NMS. The time frame of conversion needs to be furtherly investigated.

#### Dysautonomia

##### Clinical Evaluation and Rating Scales

Neurodegenerative synucleinopathies are frequently accompanied by autonomic dysfunction: constipation, urinary frequency, urinary incontinence, erectile dysfunction, and orthostatic blood pressure drop.

Patients with iRBD experience significantly more gastrointestinal, urinary, and cardiovascular functioning problems in comparison to healthy controls (50, 60, 61, 65) and frequency and severity of constipation were reportedly similar to PD patients in one study (61). Systolic blood pressure drop was found to be significantly higher in PD patients with RBD than in those without RBD in an earlier study (50).

In a prospective observational study comparing 32 converted RBD subjects versus 59 non-converted patients (66), autonomic dysfunction evaluated by the Unified Multiple System Atrophy Rating Scale (UMSARS) was more prominent in patients who had RBD with defined synucleinopathy than in iRBD subjects. Even more systolic drop, erectile dysfunction, and constipation could identify disease conversion up to 5 years in advance, with sensitivity ranging from 50 to 90% (66).

In a multicenter study of iRBD subjects, the 93 convertors scored significantly higher on SCOPA-AUT than the 186 non-convertors (14.1 ± 6.1 vs 12.0 ± 6.9) (67). The differences were primarily related to increases in the gastrointestinal and cardiovascular domains, with an additional statistically non-significant increase in urinary symptoms.

Fereshtehnejad et al. (63) calculated the risk of conversion to PD/DLB in their longitudinal study of iRBD subjects based on MDS research criteria for prodromal PD, including assessment of constipation, erectile dysfunction, urinary dysfunction and orthostatic hypotension (see Table 4). The prodromal criteria had 81.3% sensitivity and 67.9% specificity for conversion to PD/DLB at 4-year follow-up, while one year before conversion, sensitivity was 100%. The MDS criteria predicted conversion to DLB with even higher accuracy than to PD without dementia at disease onset.

**Table 4:** Predictive value of autonomic symptoms for phenoconversion in idiopathic RBD subjects.

Study	Study population	AUTONOMIC SYMPTOMS			
Postuma et al. (67)	Non-convertors *n* = 169 vs convertors *n* = 72 (PD = 39, Possible DLB = 47, MSA = 7)		SCOPA – AUT* total		
			11.97 ± 6.93 vs 14.07 ± 6.05		
			Adjusted OR (95% CI) 1.13 (1.01–1.25)		

Li et al. (31, 64)	Non-convertors *n* = 25 vs Convertors *n* = 18 (DLB = 2, MSA = 3, PD/mild cognitive impairment = 4, and PD = 9)	CONSTIPATION Response: Yes	SCOPA – AUT** ≥ 11	NMSQ ≥ 12	
		56 vs 66.7%	20 vs 66.7%	8 vs 33.3%	
		HR (95% CI, adjusted) 1.48 (0.55–3.98)	HR (95% CI, adjusted) 4.46 (1.64–2.10)	HR (95% CI, adjusted) 3.11 (1.15–8.40)	

Fereshtehnejad et al. (63)	Non-convertors *n* = 73 Convertors *n* = 48 (PD/DLB)	CONSTIPATION* UMSARS score ≥ 2	Erectile dysfunction* (only men) UMSARS score ≥ 3	Urinary dysfunction UMSARS score ≥ 2	Orthostatic hypotension** UMSARS score ≥ 2
		19.1 vs 31.2% +LR 1.63 (0.87–3.06)	30.4 vs 44.1% +LR 1.45 (0.84–2.51)	2.7 vs 8.3% +LR 3.04 (0.58–15.96)	23.3 vs 45.8% + LR 1.97 (1.17–3.30)

*PD, Parkinson’s disease; DLB, dementia with Lewy Body; MSA, multiple system atrophy; SCOPA-AUT, Scale for Outcomes in Parkinson Disease—Autonomic; OR, odds ratio; CI, confidence interval; NMSQ, Non-Motor Symptom Questionnaire; HR. hazard ratio; UMSARS. Unified Multiple System Atrophy Rating Scale; Orthostatic hypotension tested by manual measurement of blood pressure in supine position and after 1-min standing; +LR, positive likelihood ratio*.

*p < 0.05; **p < 0.01; ***p < 0.001.

The recent study by Li et al. (64) confirmed that patients with higher scores on the NMSQuest and SCOPA-AUT were more likely to develop synucleinopathy (see Table 4).

##### Conclusion

Questionnaire-assessed autonomic dysfunction is significantly more frequent and severe in iRBD compared to controls. Orthostatic drop, erectile dysfunction, and constipation independently increase the LR for the conversion to synucleinopathy. There is not enough evidence to support the predictive role of urinary dysfunction in iRBD subjects specifically. Severity of autonomic dysfunction in iRBD and PD patients was found comparable in some of the studies.

##### Electrophysiological Testing and Imaging

Cardiac autonomic testing during wakefulness showed that only 5/14 patients (36%) with RBD (10 iRBD) had normal results in all traditional autonomic tests during wakefulness, and all had reduced heart rate variability (HRV) during sleep (68). While reduced nocturnal HRV generally correlated with impairment while awake, patients with normal autonomic activity during wakefulness showed reduced nocturnal HRV. Another study also (69) found significant differences in HRV between iRBD and healthy control subjects; however, the investigators did not find a difference between those who converted to PD, MSA or DLB and those who did not. Also, RR interval, HRV, and respiratory frequency remained stable in RBD subjects in comparison with healthy controls when evaluated from stage 2 NREM to REM sleep (70). Sorensen et al. (71) found an attenuated sympathetic nervous system activity in RBD patients (11 iRBD, 14 PD patients with RBD) based on HRV evaluation compared to controls. The HRV pathology was even more pronounced in PD patients compared to iRBD. Another study (72) analyzed heart rate response to arousals or to leg movements during sleep in PD patients with RBD or without RBD, iRBD subjects and healthy controls. The heart rate responses for the iRBD group were intermediate with respect to the control and PD groups. Frauscher et al. (40) showed that, in a cohort of 15 iRBD subjects, 15 controls, and 15 PD patients: (a) blood pressure changes in the tilt table examination were similar between iRBD patients and healthy controls, but blood pressure drops were more pronounced in PD; (b) in the orthostatic standing test, iRBD patients had higher blood pressure changes than healthy controls and highest drops were found in the PD group; (c) Valsalva ratio was lower in iRBD and PD compared to healthy controls; and (d) total COMPASS score was higher in iRBD compared to healthy controls, with highest scores found in PD.

##### Conclusion

Heart rate variability, peripheral sympathetic system activity, autonomic responses to arousal and leg movements in sleep and orthostatic testing show higher prevalence and severity of autonomic dysfunction in iRBD subjects compared to healthy controls. Autonomic evaluation during sleep could identify impaired cardiac dysautonomia in RBD patients earlier than the traditional tests during wakefulness. There is insufficient evidence to calculate diagnostic accuracy of electrophysiological testing in prediction of phenoconversion to manifest synucleinopathy.

#### Daytime Sleepiness

Excessive daytime sleepiness (EDS) is associated with the later stages of PD and there are studies documenting higher risk of PD in men reporting daily napping (73). Arnulf et al. (74) showed that the 75 patients with iRBD in their study were sleepier (Epsworth Sleepiness Scale (ESS): 7.8 ± 4.6) at the time of RBD diagnosis than 74 age- and sex-matched controls (ESS: 5.0 ± 3.6, *p* < 0.0001). Those who had ESS scores greater than 8 at the time of RBD diagnosis had higher probability to convert to parkinsonism and dementia, both from RBD onset and from RBD diagnosis within the follow-up of 1–15 years (median 3 years) (74). A more recent study investigated 179 patients with iRBD (75). Of these, 45 patients with ESS score ≥ 14 were defined as having EDS. After a mean follow-up of 5.8 years (SD = 4.3 years), 50 (27.9%) patients developed neurodegenerative diseases. There was a significantly higher proportion of conversion in patients with EDS compared to those without EDS (42.2 vs 23.1%, *p* = 0.01). EDS significantly predicted an increased risk of developing neurodegenerative diseases (adjusted HR = 2.56, 95% CI 1.37–4.77) after adjusting for age, sex, body mass index, current depression, OSA, and PLM. Further analyses demonstrated that EDS predicted the conversion to PD (adjusted HR = 3.55, 95% CI 1.59–7.89) but not dementia (adjusted HR = 1.48, 95% CI 0.44–4.97) (75). Similarly, higher HR to phenoconvert has been found in iRBD subjects reporting EDS (HR 2.73, 95% CI 1.06–7.05, *p* = 0.038) based on the non-motor Symptom Questionnaire (64).

On the other hand, a recent study by Postuma et al. (76) did not find sleepiness as a predicting factor for future conversion. The same study did not reveal any difference in subjective rating on the Insomnia Severity Scale in convertors and non-convertors (76).

##### Conclusion

Excessive daytime sleepiness seems to be more common in iRBD subjects compared to controls. EDS seems to increase the likelihood of phenoconversion to synucleinopathies, although this has not been confirmed in all studies. Further evidence is needed to confirm diagnostic accuracy and time frame of phenoconversion. There is insufficient evidence to consider insomnia as a symptom indicating higher risk of phenoconversion.

#### Depression, Anxiety

A case–control study (77) showed that patients with iRBD were more likely to report comorbid depression based on questionnaire assessment than controls (OR 2.0, 95% CI 1.3–2.9). Depression was found to be associated with reduced ability to recall enacted dreams (32). No differences in depressive symptomatology were found between iRBD and healthy controls evaluated by the Hospital Anxiety and Depression Scale (78). Also, no differences were found between depressive symptomatology evaluated in two recent studies (63, 64) by the Hamilton Depression Rating Scale and Beck Depression Inventory in the RBD convertors and non-convertors. It was suggested that antidepressants unmask latent RBD rather than cause it, in a study in which medications were modified, RBD symptoms improved, but the RSWA persisted (79).

A prospective cohort study of anxiety predictors showed more symptoms of anxiety at baseline that increased over time in patients with RBD compared to early stage untreated PD (80).

##### Conclusion

Depressive symptoms do not identify RBD subjects with the risk for conversion to synucleinopathy. There is not enough evidence for depression and anxiety to be considered risk factors for neurodegeneration in RBD patients.

#### Color Vision Impairment

The abnormality of color vision is one of the NMS in patients with PD, and the severity of axial motor symptoms is closely related to visual dysfunction (81).

In a 10-year prospective study of an iRBD cohort over the age of 55 years old, color vision deficit (Farnsworth-Munsell 100-hue test) increased the HR of neurodegenerative disease conversion by 3.1-fold (49). Color vision in iRBD was reduced as much as 5 years before synucleinopathy diagnosis, with only slight decline in preclinical stages (82).

##### Conclusion

Color vision might be helpful in predicting phenoconversion in iRBD subjects. The data are, however, limited and need confirmation in further studies.

#### Cognitive Impairment

Significant differences in cognitive functioning (memory, executive functions) between iRBD and healthy controls have been found (83–86). The frequency of mild cognitive impairment (MCI) was estimated to be 33–50% in iRBD patients and 63–73% in PD patients with RBD, respectively (87). The high conversion rate of iRBD was interconnected with a 2.2-fold increase in risk of developing MCI in a population-based sample (88). MCI status in subjects with iRBD strongly predicts conversion to dementia related to synucleinopathy (85, 89), but not PD based on a 10-years follow-up study of 89 subjects with iRBD (49). Cognitive tests assessing attention and executive functions strongly predict conversion to dementia in iRBD patients (89), Cross-sectional studies indicate that a proportion of iRBD patients show a pattern of cognitive deficits similar to those typically found in patients with synucleinopathies, especially in LBD or PD with MCI or PD with dementia (85, 89–91). In iRBD selective attention, executive functions and episodic verbal and non-verbal memory are the most affected (83–85, 92). Positive correlation between time-based prospective memory performance and reduced striatal dopamine transport uptake on DaT scan on the more affected side has been found (86), indicating that impaired prospective memory may also be a marker for neurodegeneration associated with dopamine depletion syndromes. However, the presence of visuospatial and visuoconstructional deficits has also been reported in some studies (92–94), especially in those iRBD subgroups that later convert to PD dementia or DLB.

##### Conclusion

Cognitive impairment is a sign of future phenoconversion but there is not enough evidence to recommend optimal set of neuropsychological examinations for precise estimation of the disease evolution.

### Neuroimaging Biomarkers

#### Presynaptic Dopaminergic Imaging

Imaging of the nigrostriatal dopaminergic pathway typically consists in measuring dopamine transporter (DAT) density (95) and positron emission tomography (PET) and single-photon emission computed tomography (SPECT) are used in this indication.

Presynaptic PET and SPECT have been shown to identify PD and related disorders in very early stages. Striatal DAT uptake has been shown to decrease with expected pattern of disease progression from healthy controls to RSWA on PSG to manifest RBD to PD (96). Compared with controls, iRBD patients had significantly reduced mean (97) I-FP-CIT binding in all four striatal regions at baseline and after 3 years. After adjustment for baseline (97) I-FP-CIT uptake ratios, the decline in Ref. (97) I-FP-CIT binding was significantly greater in patients than in controls in the left putamen. The three subjects displaying the lowest tracer uptake at baseline in this iRBD cohort were subsequently diagnosed with PD (98). The 5-year follow-up study showed that patients with lower DAT uptake (99mTc-TRODAT-1 binding) in the putamen exhibited a shorter progression-free survival time compared to the population with higher DAT uptake (64). PET using the tracer 6-[18 F] fluoro-meta-tyrosine showed that putamen and caudate uptake was significantly lower in iRBD patients than in controls (99). A recent study also revealed that a reduction of FP-CIT uptake in the putamen greater than 25% discriminated patients with DAT deficit who developed synucleinopathy from patients with DAT deficit that remained disease-free after 3-year follow-up (100). At 5-year follow-up, DAT-SPECT had a 75% sensitivity, 51% specificity, 44% PPV, 80% NPV, and a LR of 1.54 in predicting synucleinopathy. The above mentioned studies and other available data indicate that imaging of presynaptic dopaminergic integrity is suitable to monitor disease progression and identify individuals at risk of phenoconversion (101).

#### Metabolic Imaging

18F-fluorodeoxyglucose PET shows altered RBD-related regional cerebral metabolism. Increased metabolism has been found in the pons, thalamus, medial frontal and sensorimotor areas, hippocampus, supramarginal and inferior temporal gyri, and posterior cerebellum, and decreased activity in occipital and superior temporal regions (102). In another study, elevated metabolism was found in the hippocampus/parahippocampus, cingulate, supplementary motor area, and pons, and decreased metabolism in the occipital cortex/lingual gyrus (103). The above mentioned elevation in iRBD decreased with disease progression (102). Regional metabolic abnormalities were associated with clinical measures such as RBD duration and chin electromyographic activity (103).

An elevated PD-related covariance pattern in resting-state metabolic brain imaging with 18F-fluorodeoxyglucose PET has been found in subjects with iRBD compared to healthy controls. For individual subjects with RBD, final phenoconversion status has been predicted using a logistic regression model based on a PD-related covariance pattern expression and subject age at the time of imaging (104).

Resting-state functional magnetic resonance imaging (rs-fMRI) may detect preclinical alterations in brain network functioning. Connectivity measures within resting-state networks differentiated both iRBD and PD from controls, indicating its potential as an indicator of early basal ganglia dysfunction. iRBD was indistinguishable from PD on rs-fMRI despite obvious differences on DAT-SPECT. Basal ganglia connectivity is a promising biomarker for the detection of early basal ganglia disturbance and may help to identify patients at risk of developing PD in the future (105).

#### Transcranial Sonography (TCS)

Hyperechogenicity of the substantia nigra (SN) on TCS is reported in the vast majority of PD patients and only in 10% of healthy subjects. Several studies have applied TCS in iRBD and SN hyperechogenicity was found in about 35–55% of iRBD subjects and in about 70% of iRBD patients with comorbid depression (101).

Longitudinal follow-up studies showed that 36% of iRBD subjects with SN hyperechogenicity at baseline phenoconverted within 2.5 years (106) and 42% converted within 5 years (107). However, 12% iRBD patients with normal SN echogenicity at baseline also developed a neurodegenerative disorder within 2.5 years (106), and 34% within 5 years (107).

Combining TCS with other examinations might increase accuracy of prognosis. Combination of TCS with FP-CIT-SPECT revealed 100% sensitivity and 55% specificity to predict subsequent phenoconversion within 2.5 years (106). Assessment of hyposmia and mild motor impairment may also enhance the predictive value of TCS (59, 108, 109). TSC SN hyperechogenicity might act as a very weak marker for further neurodegeneration. In combination with other markers its predictive value is higher (101).

#### Cardial Imaging

In an early study by Miyamoto et al., cardiac 123I-MIBG uptake was compared in PSG confirmed iRBD subjects (*N* = 31), PD (*N* = 26), DLB (*N* = 6), and controls (110). The findings were similar among iRBD, PD and DLB, but differed from those with tauopathies and MSA. In PD patients, cardial MIBG uptake as determined by heart-to-mediastinum ratios was reduced in subjects with clinical RBD compared to subclinical RBD and those with normal REM sleep (34).

##### Conclusion

Among the available imaging methods currently available today, the measurement of presynaptic DAT density is the most informative for synucleinopathy progression evaluation (101). The HR of conversion in iRBD patients with reduced uptake on DAT-SPECTs is 3.2 higher than in subjects with normal DAT Scans.

### Electrophysiology

#### EEG

In iRBD subjects, subtle changes found in EEG during sleep (111) and wakefulness (112–114) suggest abnormalities. EEG slowing, according to a follow-up EEG study in iRBD, seems a promising marker of neurodegeneration in iRBD patients (114).

#### Polysomnography

A large and long-lasting Montreal follow-up study has found that iRBD convertors had a modest decrease in % of REM sleep (15.8 ± 8.0 vs 19.8 ± 7.5%, *p* = 0.005) at the time of diagnosis. Also, patients who converted had higher tonic REM% (58.4 ± 27.0 vs 46.1 ± 30.4%, *p* = 0.019), without any difference in phasic REM% (35.5 ± 17.0 vs 34.7 ± 18.0%, *p* = 0.81). On Cox regression analysis, adjusting for age and sex, having tonic REM > 50% was associated with a HR of 1.88 for development of neurodegenerative disease (*p* = 0.039) (76).

##### Conclusion

These electrophysiology results are interesting pathophysiologically but, to date, do not have a role for predicting phenoconversion of an individual subject with iRBD.

### Tissue Biomarkers

#### Genetics

Several gene mutations and single nucleotide polymorphisms (SNPs) have been studied in relation to iRBD. The OR for the GBA p.E326K mutation carriers to have probable RBD (RBDSQ score ≥ 6) was 3.13 in one study (*p* = 0.039) (115), while the difference in GBA mutation frequency between patients with iRBD (PSG proven) and controls showed boarderline significance (*p* = 0.05) in another recent investigation (61). On the other hand, LRRK2 mutations were not found in two iRBD cohorts (61, 116). The APOE ε4 allele frequency was similar among RBD patients and controls and it was not associated with conversion from RBD to DLB or other synucleinopathies (117). Melanoma gene variant MC1R p.R160W and other variants do not increase susceptibility for PD or RBD (118). As far as SNP studies, three SNCA-3′UTR SNPs (rs356165, rs3857053, rs1045722) were found more frequently in PD patients (with or without RBD) than in iRBD subjects (*p* = 0.014, 0.008, and 0.008, respectively) (119). In another study (120) evaluating nine PD-related SNPs, the SCARB2 rs6812193 (OR = 0.67, 95% CI = 0.51–0.88, *p* = 0.004) and the MAPT rs12185268 (OR = 0.43, 95% CI = 0.26–0.72, *p* = 0.001) were associated with RBD in different models. Kaplan–Meier survival analysis in a subset of RBD patients (*n* = 56), demonstrated that homozygous carriers of the USP25 rs2823357 SNP had progressed to synucleinopathies faster than others (log-rank *p* = 0.003, Breslow *p* = 0.005, Tarone-Ware *p* = 0.004).

##### Conclusion

There is insufficient evidence to support the role of genetic testing in identifying subjects at higher risk of RBD or for predicting phenoconversion to synucleinopathies.

#### Fluid Biomarkers

Only a limited number of studies evaluating serum and cerebrospinal fluid (CSF) biomarkers in RBD subjects have been published. Anderson et al., (121) found normal hypocretin levels in the CSF of RBD subjects. Elevated expression of prion protein (PrP, both mRNA and protein) in PD patients with RBD, compared to PD patients without sleep problems and healthy controls has been recently reported (122). Although total alpha-synuclein was reportedly lower in early untreated PD patients compared to healthy controls and CSF total tau was slightly higher, the rates of changes were not significant after 24 months of follow-up and, moreover, these CSF parameters were not studied in iRBD subjects specifically so far (123).

As far as serum biomarkers, hypercholesterolemia is possibly a predictor of lower conversion rate in iRBD according to one follow-up study (67) and higher plasma urate levels have previously been associated with a longer duration of RBD without converting to PD (97).

##### Conclusion

There is insufficient evidence to support the use of CSF and serum biomarkers as predictors for iRBD conversion to synucleinopathy.

#### Peripheral Nerve Tissue Biopsies

Parkinson’s disease-related Lewy pathology is typically present in several peripheral tissues (124, 125), some of which, like the salivary glands, colon, or skin, are readily accessible for biopsies and histopathological evaluation. Several reports have shown presence of pathological a-synuclein aggregates in peripheral tissues biopsies several years prior to onset of first motor symptoms (126, 127), raising the possibility of using these evaluations for *in vivo* confirmation of Lewy pathology in patients with iRBD. A single study has evaluated the presence of 129-phosphorylated a-synuclein (p-a-syn) in colonic biopsies, with positive findings in 4/17 (24%) iRBD patients and none of the healthy controls, resulting in excellent specificity, but low sensitivity (128). P-a-syn pathology was found only in the colonic submucosa, which is more difficult to obtain with routine colonoscopies compared to colonic mucosa. Significantly higher prevalence of colonic p-a-syn positivity in PD patients with RBD compared to patients without RBD (18/28 vs 2/15, *p* < 0.01) was reported in another study (129). Vilas et al. (130) evaluated the presence of p-a-syn in submandibular glands of 21 iRBD subjects and 26 controls with excellent specificity and good sensitivity when comparing iRBD versus healthy controls (8/9 vs 0/26 with available submandibular gland tissue). However, the needle core biopsies missed the submandibular gland in 12/21 iRBD patients even under ultrasound guidance. Two studies evaluated the presence of p-a-syn in skin with positive findings in 9/12 and 10/18 iRBD patients respectively, while none of the controls had p-a-syn findings (131, 132), thus yielding excellent specificity and moderate-acceptable sensitivity. The presence of pathological p-a-syn aggregates was more common in proximal than distal body segments. Reduced intraepidermal nerve fiber density in patients with iRBD compared to healthy controls has also been reported (133).

##### Conclusion

Although peripheral tissue biopsies seem to be a promising technique for confirmation of peripheral Lewy pathology in iRBD patients, there are several technical and methodological issues which need to be addressed prior its use in routine practice, including optimal immunostaining methodology, target regions and sampling technique among others. Moreover, there are no longitudinal studies to date evaluating the real rates of phenoconversion from iRBD with, or without, peripheral Lewy pathology to clinically manifest synucleinopathy. Thus the predictive value of this examination is still unknown and needs to be determined.

## Discussion and Conclusion

Cumulative evidence strongly supports the role of iRBD as one of the most important clinical markers of synucleinopathies. While the diagnosis is based on expensive and scarcely accessible PSG confirmation, a high need for easily available and accurate identification of RBD at risk subjects in the general population will arise with emerging therapeutic strategies, such as immunization therapies (134) and potential neuroprotective agents, including exenetide (135) and others. Also, in order to initiate this type of trials in iRBD subjects and ensure their accurate evaluation, clinical markers assessing disease progression and estimate of time of conversion to manifest synucleinopathy need to be determined. Several validated questionnaires and screening measures for RBD have been developed to enable easier identification of subjects with RBD in large-scale studies and in individual subjects. Although the diagnostic accuracy of RBD rating scales has been shown acceptable to excellent in most validation studies, caution should be exercised when interpreting these results. Most of the validation studies for RBD scales were performed in patients with a confirmed diagnosis of RBD, who were more likely to recognize and be aware of their RBD symptoms. In fact, diagnostic accuracy depends on the setting in which the questionnaire has been applied. In a study, where RBDSQ was administered to PD patients with a confirmed diagnosis of RBD and to PD patients where RBDSQ was administered as first line screening without prior sleep interview and PSG, the diagnostic accuracy was significantly lower in the latter group with only moderate sensitivity and specificity to identify the RBD subjects (30). Also, uncritical use of RBD-screening instruments may result into false-positive findings, e.g., in patients with other sleep disorders such as OSA or non-REM parasomnias, RLS, or in false negative results, as in patients who lack a bed partner and may not be aware of their RBD symptoms. Up to 16% of patients who are later considered negative on sleep interview and PSG may score positive in RBD questionnaires (136). Moreover, several studies have shown that using different RBD questionnaires in the same population yields a variable prevalence of RBD (137). Therefore, questionnaire-based diagnosis of RBD is insufficient and should be considered as probable only, with necessity of further PSG confirmation. More effective strategies for identification of RBD subjects in large populations may be based on multistep screening approaches (138, 139), using single screening questions, followed by one of the specific RBD rating scales and a more detailed sleep interview. Also, while there is no solid evidence on use of wearable technologies such as smartphones and smartwatch in screening for RBD, with advancing technologies it is likely these may become a reasonable first line screening option for RBD in the future and thus should be explored in more details.

From the clinimetric point of view, the only rating scale which has been shown to be reliable, valid (with exception of convergence validity) and sensitive to change is the RBDQ-HK. The remaining scales were either not studied appropriately from the clinimetric point or view, or the results were not satisfactory. These limitations mostly regard the validity of the scales and their sensitivity to change.

Several clinical, imaging and electrophysiological progression markers have been identified in iRBD subjects. For some of these markers, like subtle motor impairment, time from deviation of normal values to a diagnosis of synucleinopathy has been estimated in the range of 4.2–9.8 years. Several other promising markers of phenoconversion, such as olfactory loss, autonomic dysfunction, cognitive impairment, imaging markers, including presynaptic dopaminergic nuclear medicine imaging, rs-fMRI or TCS, as well as peripheral nerve tissue biopsies have been identified. Although these markers seem to predict future conversion to synucleinopathies, reported results have not always been uniform, and there is not enough evidence to support a specific set of evaluations or procedures for precise estimation of disease conversion or progression. Moreover, it is very likely that a single marker will not be sufficient to predict future conversion of iRBD to a manifest synucleinopathy and that a combination of different clinical, imaging and other markers, such as confirmation of presence of pathological a-synuclein aggregation in peripheral tissues, will be necessary for accurate estimation of future disease progression. In this regard, the recently published MDS research criteria for prodromal PD (11) have yielded sensitivity of 81.3% and specificity of 67.9% for iRBD conversion to PD/DLB at 4-year follow-up, with a 100% sensitivity 1 year before conversion (63).

Future studies are needed in order to predict conversion of iRBD into specific types of synucleinopathies. Moreover, prospective studies are necessary to confirm and explore the role of individual prodromal markers and their combination in predicting conversion from iRBD to PD, DLB, or MSA.

## Author Contributions

All authors made substantial contributions to the conception of the work, drafted sessions of the manuscript or revised it critically for important intellectual content, gave final approval of the version to be published, and agree to be accountable for all aspects of the work.

## Conflict of Interest Statement

The authors declare no financial disclosures or other conflicts of interest in regards to this manuscript. The handling Editor declared a past-authorship with one of the authors SM.
